# Enhancement of student perceptions of learner-centeredness and community of inquiry in flipped classrooms

**DOI:** 10.1186/s12909-018-1347-3

**Published:** 2018-10-23

**Authors:** Young Hwan Lee, Kyong-Jee Kim

**Affiliations:** 1 Department of Medical Humanities, College of Medicine, Yeungnam University, Daegu, South Korea; 2 Department of Medical Education, School of Medicine, Dongguk University, 32 Dongguk-ro, Ilsandong-gu, Goyang-si, Gyeonggi-do, Goyang, 10326 South Korea

## Abstract

**Background:**

Flipped classrooms (FC) are expected to create a more student-centered, active learning environment than traditional lectures. This study assessed the effectiveness of FC in promoting a student-centered, active learning environment by examining the changes in student perceptions of the learning environment of FC in terms of student-centeredness and sense of community of inquiry (COI), which represents the elements of a successful learning environment.

**Methods:**

Questionnaires were given to a cohort of Year 2 students in a six-year undergraduate medical education program, who had taken an introduction to medicine course in a FC format. The questionnaire included 7 items on the teaching orientation (i.e., teacher-centered vs. student-centered) and 34 items from the Community of Inquiry Survey Instrument, which consisted of three sub-scales – teaching, social, and cognitive presences. The questionnaires were administered in pre- and post-test format during the first and last week of the semester.

**Results:**

A total of 55 students returned the questionnaires (100% response rate). The student perceptions of student-centeredness of FC increased significantly over time (*p* < 0.001), and their perceptions of COI in FC also increased significantly in all three presences (*p* < 0.001). The student perceptions of student-centeredness and sense of COI in FC increased significantly in both high-achieving and low-achieving students (*p* < 0.01).

**Conclusions:**

This study indicates that the flipped classroom model is an effective approach to fostering a learner-centered learning environment and developing a community of inquiry among medical students.

## Background

With the rapid increase in medical knowledge nowadays, there have been concerns over passively transferring knowledge to learners using traditional lectures in medical education. Hence, there is increasing pressure for medical education to move toward more student-centered, active learning [1–3]. The fourth industrial revolution and the rapidly changing technologies call for reforms in how medical students learn so they can keep up with the healthcare environment for the coming decades. In doing so, medical schools need to offer learning environments that promote students’ active learning to help them develop the competencies required for tomorrow’s doctors. Accordingly, there has been increasing attention to the flipped classroom model to create a more student-centered learning environment in medical education [1–3].

In flipped classrooms, which is also referred as the inverted classroom model [4], the in-class and out-of-class activities in conventional lectures are ‘flipped,’ in which students acquire knowledge by engaging in pre-assignments by using the learning contents are delivered to the students using technology, such as video lectures, before they meet in the classroom, and then they engage in active learning in the classroom instead of lectures [5]. Evidence indicating the effectiveness of flipped classrooms in higher education has been provided [6], and there is a growing body of literature in medical education indicating that flipped classrooms is an effective approach to learning and teaching [7–12].

Still, a systematic review study of flipped classrooms in medical education shows there is no solid evidence yet about the effectiveness of the flipped classroom [13]. Moreover, previous studies have focused on knowledge acquisition, and there is little information on student perceptions of the effectiveness of its learning environment. Active learning approaches, like flipped classrooms, can cultivate a student-centered learning environment that promotes collaborative interaction for deep and meaningful learning [14]. Such learning experiences are fundamental for building the capacity for lifelong learning [15], which are necessary skills for tomorrow’s doctors [16]. Still, there is little evidence showing that the students’ experience with flipped classrooms contributes to building such learning environments. This study examined the effectiveness of flipped classrooms in promoting a student-centered, active learning environment that fosters collaborative interaction for deep and meaningful learning.

## Methods

### The study participants and setting

The study participants were a cohort of 2nd year students (*N* = 55) in the six-year basic medical education program at a private urban medical school in South Korea. The students were enrolled in an introductory medicine course in the fall of 2017. This course was designed in a flipped classroom model with the aim of promoting student-centered learning. This was a two-credit hour course offered for a duration of 15 weeks throughout the semester and covered a variety of ethical or social issues in the practice of medicine. Out of a total of 30 h of in-class instruction time in this course, 24 h (80%) were delivered in flipped classroom format and the remaining six hours were taught by conventional lectures.

In this flipped classroom format, the students were given pre-class assignments of reading materials and video lectures that would help them acquire knowledge relevant to the issues to be covered in the class. Students spent 1–3 h in these out-of-class activities for each flipped classroom unit at their own pace in preparation for in-class activities. During the class, the students took quizzes on the knowledge acquired from the out-of-class activities and then debated on the issues using the classroom debate method. In this classroom debate, students were assigned to eight teams and two teams participated in a structured academic debate in each session. Those two teams were assigned to present the pro or con and to research background information about the issues and the other students assessed the debate teams as the audience. At the end of the debate, the students submitted reports that summarized how their opinions had changed after the debate. The report also included student self-assessment of his or her overall performance in the debate.

### Study design and procedures

A questionnaire was designed and implemented to investigate the participants’ perceptions of the learning environment in flipped classrooms. This questionnaire consisted of three sections: (1) participant demographics, (2) teaching orientation, and (3) Revised Community of Inquiry.

The ‘teaching orientation questionnaire’ was developed by Kim et al. [17] and consisted of 7 items to assess the extent to which the classroom activities are student-centered. This instrument used a bipolar scale to rate the design and implementation of the classroom activities, which ranged from teacher-centered (highly structured, direct instruction) to student-centered (mostly unstructured, open-ended learning) [17]. Kim et al. [17] reported that the reliability score of the teaching orientation questionnaire was 0.72. This instrument was translated into Korean by the authors and pilot tested with three premed medical students to evaluate the clarity of the items. They were asked to think aloud while they completed the questionnaire and to make comments on any words or phrases not clear or unfamiliar to them. The students commented there were no items unclear for them.

The items in the third section in the questionnaire for this study were adapted from the ‘Revised Community of Inquiry’ instrument [18, 19]. This instrument is based on the theoretical framework of the community of inquiry, which represents elements of a successful learning environment by creating a collaborative-constructivist learning experience [18]. This framework was comprised of three elements - teaching, social, and cognitive presence. The teaching presence refers to the design and facilitation of learning experiences by the instructor. Social presence refers to the level of interaction and cohesiveness of the participants in the class. Cognitive presence is the extent to which students can construct meaning through sustained communication. Based on this framework, the Revised Community of Inquiry instrument consisted of 34 items: 13 items on teaching presence, 9 on social presence, and 12 on cognitive presence. Those items were drawn from 10 sub-scales, three on the teaching presence (design & organization, facilitation, and direct instruction), three on the social presence (affective expression, open communication, and group cohesion), and four on the cognitive presence (triggering event, exploration, integration, and resolution). This instrument has been used in many studies across various settings and found to be valid and reliable [20]. A Korean version of the Community of Inquiry instrument translated and validated by Yu and Richardson [21] was used for this study. This Korean instrument demonstrated acceptable internal consistency, where Cronbach’s α for all three presences were 0.91 or higher [21].

The questionnaires were implemented in the pre- and post-test format during the first and last week of the semester in fall, 2017 to investigate the changes in student perceptions of the learning environment over time by comparing student perceptions assessed by the pre- and post-questionnaires. Participation in the study was voluntary and consent was implied with the return of the survey as the responses were collected anonymously. An ethical review was conducted and informed consent was exempted by the institutional review board of Dongguk University, Gyeongju (IRB approval number: DGU IRB 20170020).

### Data analysis

Reliability analysis of each component in the questionnaire was performed to check for its internal consistency. A paired t-test was conducted on the student responses in pre- and post- questionnaires to compare their perceptions of flipped classrooms over time. Moreover, the participant perceptions were compared between the groups who were high performers and those who were low achievers in terms of their cumulative GPA. The cutoff scores for categorizing the students into the high- and low- performers groups were set using their median GPAs, which was 3.0 out of 4.5. An independent sample t-test was conducted for between-group comparisons of the students’ responses between the low-performing and high-performing groups.

Statistical analysis was performed using the IBM-SPSS version 22 for Windows. A *p* value less than 0.05 was considered significant.

## Results

### Participant demographics

In total, 55 students completed both the pre- and post- questionnaire (a 100% response rate): 67% (*n* = 37) were male and 33% (*n* = 18) were female. Their ages ranged from 19 to 24 years (M = 19.76 ± 1.30).

### Reliability analysis

The Cronbach’s alpha coefficient for the seven items in the teaching orientation questionnaire was 0.79, demonstrating reliable internal consistency. The reliability for the three sub-scales in the community of inquiry instrument was also at acceptable levels, where the Cronbach’s alpha coefficients ranged from 0.81 to 0.93.

### Student-centeredness of flipped classrooms

Table 1 lists the participant responses on the teaching orientation of the flipped classrooms. The student perceptions of the student-centeredness of flipped classrooms increased significantly over time (*p* < .001). The participant perceptions of student-centeredness of flipped classrooms measured in the pre-course survey did not differ between higher- and lower-performing students (*t* = 1.18, *p* = .08), and their perceived student-centeredness increased significantly in both groups over time (*p* < .01) as seen in Table 1.

**Table 1:** Student perceptions of teaching orientation in flipped classrooms^a^

High achievers (*n* = 26)			Low achievers (*n* = 29)			Total (*n* = 55)		
M (SD)		t (*p*)	M (SD)		t (*p*)	M (SD)		t (*p*)
Pre	Post		Pre	Post		Pre	Post	
2.95 (.38)	3.64 (.57)	5.69 (<.001)	2.79 (.44)	3.26 (.50)	3.68 (<.001)	2.89 (.42)	3.43 (.56)	6.38 (<.001)

^a^1 = “teacher-centered,” 5 = “student-centered”

### Community of Inquiry in flipped classrooms

Table 2 shows participant perceptions of the community of inquiry in flipped classrooms in pre- and post-course surveys. The participants’ sense of community of inquiry increased significantly over time in all three presences. Moreover, Table 2 illustrates group comparisons of student perceptions between higher- and lower-performing students. There were no differences in student perceptions of community of inquiry in all three presences in neither pre-course nor post-course surveys between the two groups.

**Table 2:** Student perceptions of the Community of Inquiry in flipped classrooms in the pre- and post-course surveys^a^ (*n* = 55)

Sub-scales	Pre-course survey			Post-course survey			Total		
	M (SD)		t (*p*)	M (SD)		t (*p*)	M (SD)		t (*p*)
	High performers	Low performers		High performers	Low performers		Pre	Post	
Teaching presence	3.51 (.57)	3.29 (.67)	1.32 (.19)	3.69 (.66)	3.76 (.47)	.50 (.62)	3.39 (.63)	3.73 (.57)	3.70 (<.001)
Social presence	3.26 (.47)	3.41 (.62)	.95 (.34)	3.61 (.69)	3.77 (.59)	.92 (.36)	3.34 (.55)	3.70 (.64)	5.23 (<.001)
Cognitive presence	3.16 (.47)	3.12 (.57)	.29 (.77)	3.78 (.61)	3.82 (.50)	.21 (.84)	3.14 (.52)	3.80 (.55)	8.82 (<.001)

^a^1 = “strongly disagree,” 5 = “strongly agree”

**Fig. 1:**
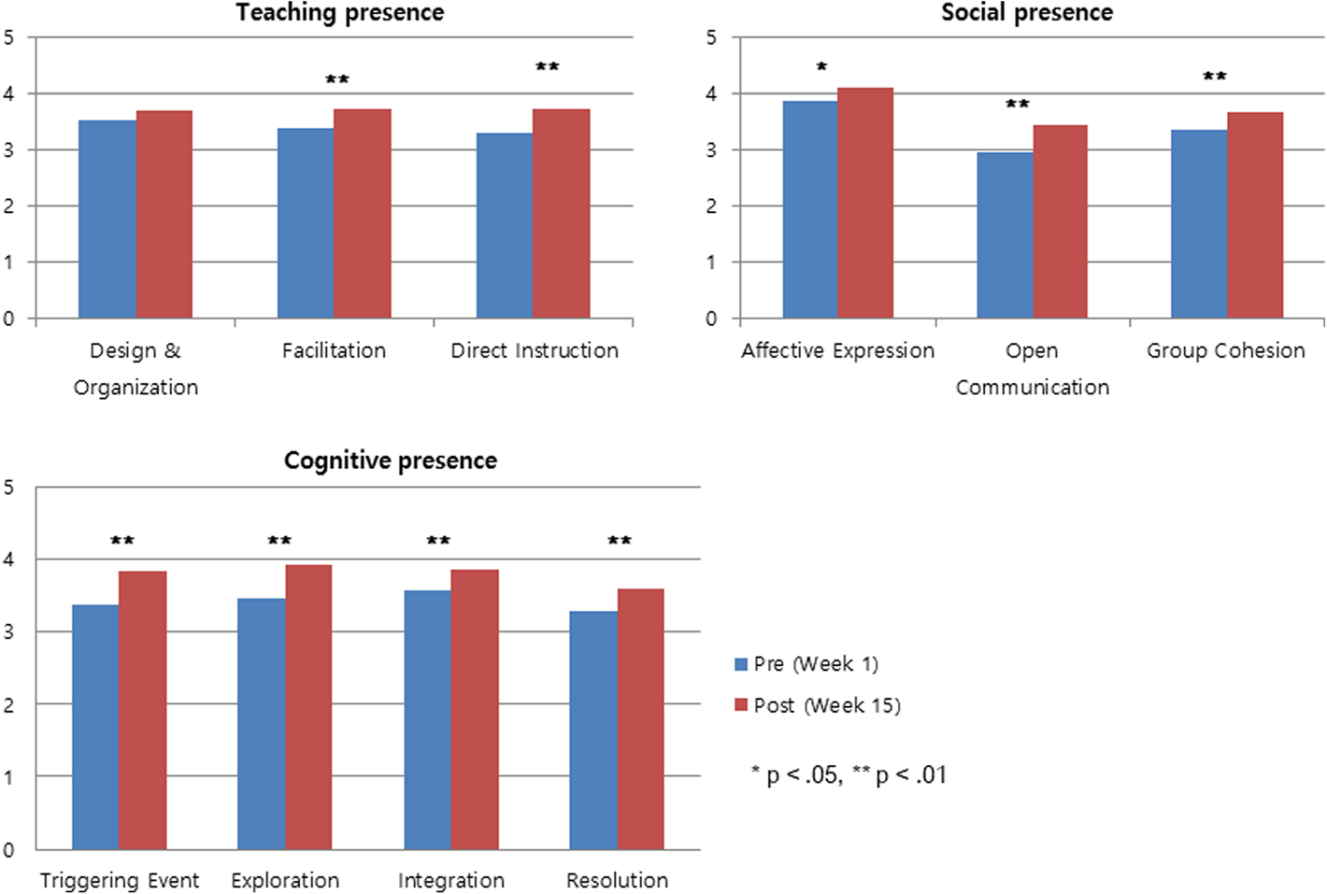
Changes in student perceived sense of Community of Inquiry in flipped classrooms (*n* = 55) ^§^. ^§^ 1 = “strongly disagree,” 5 = “strongly agree”

Figure 1 illustrates the changes in participant perceptions of the community of inquiry in flipped classrooms in each sub-scale of three presences in the community of inquiry framework. The students’ sense of community of inquiry increased significantly in nine out of 10 sub-scales of the three presences. No significant changes were observed in student perceptions in the design and organization of instruction, which was a sub-scale of the teaching presence (*t* = 1.83, *p* = 0.07).

A within-group comparison of changes in student perceptions of community of inquiry in flipped classrooms observed in higher and lower performing groups is provided in Table 3. Significant changes were observed in both higher and lower performing students in terms of their sense of social and cognitive presences in flipped classrooms (*p* < 0.01). No significant change in the sense of teaching presence was observed among higher performing students (*t* = 1.01, *p* = 0.32), but it increased significantly among lower performing students (*t* = 4.79, *p* < 0.001).

**Table 3:** Student perceptions of the Community of Inquiry in flipped classrooms in lower and higher performing groups^a^

Sub-scales	High-performers (*n* = 26)			Low-performers (*n* = 29)		
	M (SD)		t (*p*)	M (SD)		t (*p*)
	Pre	Post		Pre	Post	
Teaching presence	3.51 (.57)	3.69 (.66)	1.01 (.32)	3.29 (.67)	3.76 (.47)	4.79 (<.001)
Social presence	3.26 (.47)	3.61 (.69)	3.43 (<.01)	3.41 (.62)	3.77 (.59)	3.89 (<.01)
Cognitive presence	3.16 (.47)	3.78 (.61)	6.21 (<.001)	3.12 (.57)	3.82 (.50)	6.27 (<.001)

^a^1 = “strongly disagree,” 5 = “strongly agree”

## Discussion

This study shows that flipped classrooms promote student-centered learning and are conducive to developing a sense of teaching, social, and cognitive presence in the learning environment. Moreover, student perceptions of the learning environment in flipped classrooms changed more positively in both higher and lower performing students. In particular, changes in the students’ sense of teaching presence were more significant among the lower performing students than their higher performing peers. This finding suggests that flipped classrooms are more beneficial for lower performing students than higher performers in enhancing their sense of teaching presence. This finding is in line with that of other previous studies that the flipped classroom may benefit lower performing students than higher performing students in medical and college courses [22, 23].

This study had several limitations. First, this study was conducted of a relatively small sample, which may affect the generalizability of the study. Yet, it can be argued that the sample was fairly representative of demographics of Korean medical students. Second, this was not an experimental study and a convenience sampling method was used; thus, a comparison of the student perceptions of flipped classrooms with conventional lectures was not available in this study. Therefore, this study does not give direct evidence as to how effective flipped classrooms are in creating a community of inquiry compared to traditional lectures. Third, as various learning approaches are viable for in-class and out-of-class activities in flipped classrooms, student experience and perceptions of learning may differ across such learning activities. Therefore, it needs to be taken into account to what extent the student learning experience with classroom debates implemented for in-class activities in this study affected their perceptions of the learning environment compared to other in-class learning activities.

Although this study found student perceptions of the learning environment were enhanced when they experienced flipped classrooms, it did not explore what influenced these changes. Future study is warranted to analyze student perceptions by using more various sources of data, in particular using qualitative research methods, for better understanding of the factors that influenced their learning experiences. Such understanding of factors that influence students’ perceptions of teaching orientation and the development of sense of teaching, social, and cognitive presence will have implications for understanding design principles for flipped classrooms that promote a student-centered learning environment and develop sense of community of inquiry.

## Conclusions

The flipped classroom model is an effective approach to fostering a learner-centered learning environment and to developing a community of inquiry among medical students. In particular, flipped classrooms appear to have a positive influence to a greater degree on low achievers.

## Abbreviations

COI: Community of Inquiry
FC: Flipped Classrooms
